# Improved isolation strategies to increase the yield and purity of human urinary exosomes for biomarker discovery

**DOI:** 10.1038/s41598-018-22142-x

**Published:** 2018-03-02

**Authors:** Ali Hashemi Gheinani, Mike Vögeli, Ulrich Baumgartner, Erik Vassella, Annette Draeger, Fiona C. Burkhard, Katia Monastyrskaya

**Affiliations:** 1 Urology Research Laboratory, Department for BioMedical Research, University of Bern, Bern, Switzerland; 2 medi Zentrum für medizinische Bildung, Biomedizinische Analytik HF Max-Daetwyler-Platz 2, 3014 Bern, Switzerland; 3 Institute of Pathology, University of Bern, Bern, Switzerland; 4 Institute of Anatomy, University of Bern, Bern, Switzerland; 5 Department of Urology, University Hospital, 3010 Bern, Switzerland

## Abstract

Circulating miRNAs are detected in extracellular space and body fluids such as urine. Circulating RNAs can be packaged in secreted urinary extracellular vesicles (uEVs) and thus protected from degradation. Urinary exosome preparations might contain specific miRNAs, relevant as biomarkers in renal and bladder diseases. Major difficulties in application of uEVs into the clinical environment are the high variability and low reproducibility of uEV isolation methods. Here we used five different methods to isolate uEVs and compared the size distribution, morphology, yield, presence of exosomal protein markers and RNA content of uEVs. We present an optimized ultracentrifugation and size exclusion chromatography approach for highly reproducible isolation for 50–150 nm uEVs, corresponding to the exosomes, from 50 ml urine. We profiled the miRNA content of uEVs and total urine from the same samples with the NanoString platform and validated the data using qPCR. Our results indicate that 18 miRNAs, robustly detected in uEVs were always present in the total urine. However, 15 miRNAs could be detected only in the total urine preparations and might represent naked circulating miRNA species. This is a novel unbiased and reproducible strategy for uEVs isolation, content normalization and miRNA cargo analysis, suitable for biomarker discovery studies.

## Introduction

Concomitant with aging of the population, the incidence of bladder outlet obstruction-induced lower urinary tract (LUT) disorders is steadily growing, and now poses a common and recurrent problem in urological practice. Bladder outlet obstruction leads to organ hypertrophy and fibrotic remodelling, often resulting in loss of bladder contractility. Reliable markers of bladder function are urgently needed in order not to surpass the “point of no return”, leading to bladder decompensation/failure, when timing the surgical intervention. MicroRNAs (miRNAs), small non-coding single-stranded RNAs, are important modulators of gene expression, and dysregulation of their synthesis contributes to many human diseases^1,2^ making them potential biomarkers for diagnostic applications. A number of regulatory miRNAs have been implicated in bladder pathologies^3–7^ and function^8^. Increasing evidence indicates that miRNAs may play a role in the regulation of urothelial permeability^9^ and bladder contractility^4,10^. Recently, using human patients’ biopsies we completed the comprehensive transcriptome and miRNA profiling of different urodynamically-defined states of the bladder and identified three-miRNA biomarker signatures of obstruction-induced lower urinary tract dysfunction^11^.

Biopsy collection is the gold standard method for the diagnosis and monitoring of many urological disorders. However, it is an invasive procedure of restricted reproducibility. In addition, the high variability between the biopsy samples of the same patient is an important drawback^12^. In contrast, urine is a body fluid, which is easy to collect non-invasively, making it an attractive source of potential biomarkers. It is not subjected to homeostatic mechanisms and its contents reflect many changes of the body, such as pregnancy, aging, and disease. Most studies on urinary biomarkers are linked to kidney diseases^13,14^ and cancers^15^, mostly of the urogenital system such as bladder^16,17^ and prostate cancer^17,18^; however, there have been attempts to find urinary biomarkers for other disorders including brain diseases^19^ and bladder dysfunction. Urine contains a considerable number of proteins, as well as small molecule metabolites and urinary extracellular vesicles (uEVs)^20^. Therefore, it would be an ideal body fluid for the diagnosis and monitoring of patients with upper and lower urinary tract diseases. Consequently, investigating urinary proteomes^13,14,21,22^ and transcriptomes^16,18,23^ are burgeoning fields.

The majority of studies utilize total, unfractionated urine samples, comprised of a complex mixture of salts, proteins, metabolites, cells, and cell debris. These components mostly originate from the upper and lower urinary tract^24^, but may also be filtered directly from systemic circulation as has been suggested for miRNAs^25^. The urinary composition can pose challenges for biomarker detection as systemic circulating molecules that are renally excreted can contaminate exosomes from the urinary tract. In contrast, the molecules packaged within uEVs provide a starting material, which has less complexity, and miRNAs are protected against degradation in an environment with high RNAse content^26,27^. RNAs are better preserved in urinary microvesicles compared to urinary cell isolates, suggesting that microvesicles may protect RNA during urine passage^24^. The LC-MS/MS analysis of exosomes identified proteins from various renal epithelia (APN, AQP1, AQP2) and the urinary bladder urothelium (uroplakin-1 and -2). Thus, proteomic analysis of uEVs can potentially provide an insight into the physiological or pathophysiological processes in many epithelial cell types facing the urinary tract^20^. Extracellular vesicles comprise a heterogeneous population of exosomes (40–200 nm diameter), microvesicles (ectosomes, 50–1,000 nm), apoptotic bodies (50–5,000 nm)^28,29^ or Golgi vesicles^30^. Their biogenesis differs: microvesicles bud directly from the plasma membrane, exosomes are the result of endocytosis and the formation of multivesicular bodies, and apoptotic bodies appear during apoptotic disintegration. In Urology research, attention has been focused on exosomes and their role in tumour progression, survival, invasion and angiogenesis by affecting the intercellular signalling through RNA transfer^31–33^.

Current enthusiasm for biomarker discovery has led to a lack of consistency in uEV isolation and characterization methods. The observation that EVs can shuttle functional nucleic acids (mRNA, miRNA or other RNA species) between cells has fundamentally changed our thinking about gene regulation. EVs can potentially regulate the recipient cell at the post-transcriptional level, however one needs to consider the complexity of the extracellular milieu which can harbour extracellular RNAs in different sub-types of uEVs such as ectosomes^15^ or non-EV carriers, including lipoproteins^34^. Separation of different sub-types of uEVs and non-vesicular entities from the exosomes is not achieved by common uEV isolation protocols. Technical complexity of the existing methods of uEV isolation, and the growing number of commercially available products add a new source of variability^35^. Comparisons of isolation methods often lack experimental characterization of the extracellular vesicles and their functions^36^.

To address this issue, we isolated uEVs from urine samples of healthy donors using five different methods and compared the uEV yield and size distribution, particle morphology, protein marker presence, and RNA content. Having selected the optimal conditions of uEV isolation, we proceeded with miRNA characterization by NanoString and validated the results by qPCR using Advanced TaqMan miRNA assays. Here we present novel unbiased and reproducible strategies for uEVs isolation, content normalization and miRNA cargo analysis, suitable for biomarker discovery studies.

## Results

### Experimental design

This study included three steps:

1. Comparative analysis of urinary extracellular vesicles (uEVs) isolated with five different methods. Urine samples were collected from healthy volunteers, pooled and an equal volumes processed with five different methods, the isolated uEVs were characterized for yield, purity, size distribution and vesicle morphology
2. Further optimization of the yield and purity of the uEVs fraction of the selected isolation method by investigating the effect of filtration (0.22 µm filters), addition of protease inhibitors (PI) and DL-dithiothreitol (DTT).
3. Validation of the selected isolation method by performing miRNA profiling of total urine and urinary exosomes.

First-void mid-stream urine samples were collected and processed as described in Methods. The samples were pooled and processed, then divided into 5 × 50 ml fractions used for 1) ultracentrifugation (UC), 2) polyethylene glycol precipitation (PEG), 3) protein concentration and size-exclusion chromatography (C-SEC), 4) ultracentrifugation and SEC (UC-SEC), 5) polyethylene glycol precipitation and SEC (PEG-SEC) (Fig. S1).

### Comparison of yields, size distribution and protein content of uEVs isolated with different methods

**Figure 1:**
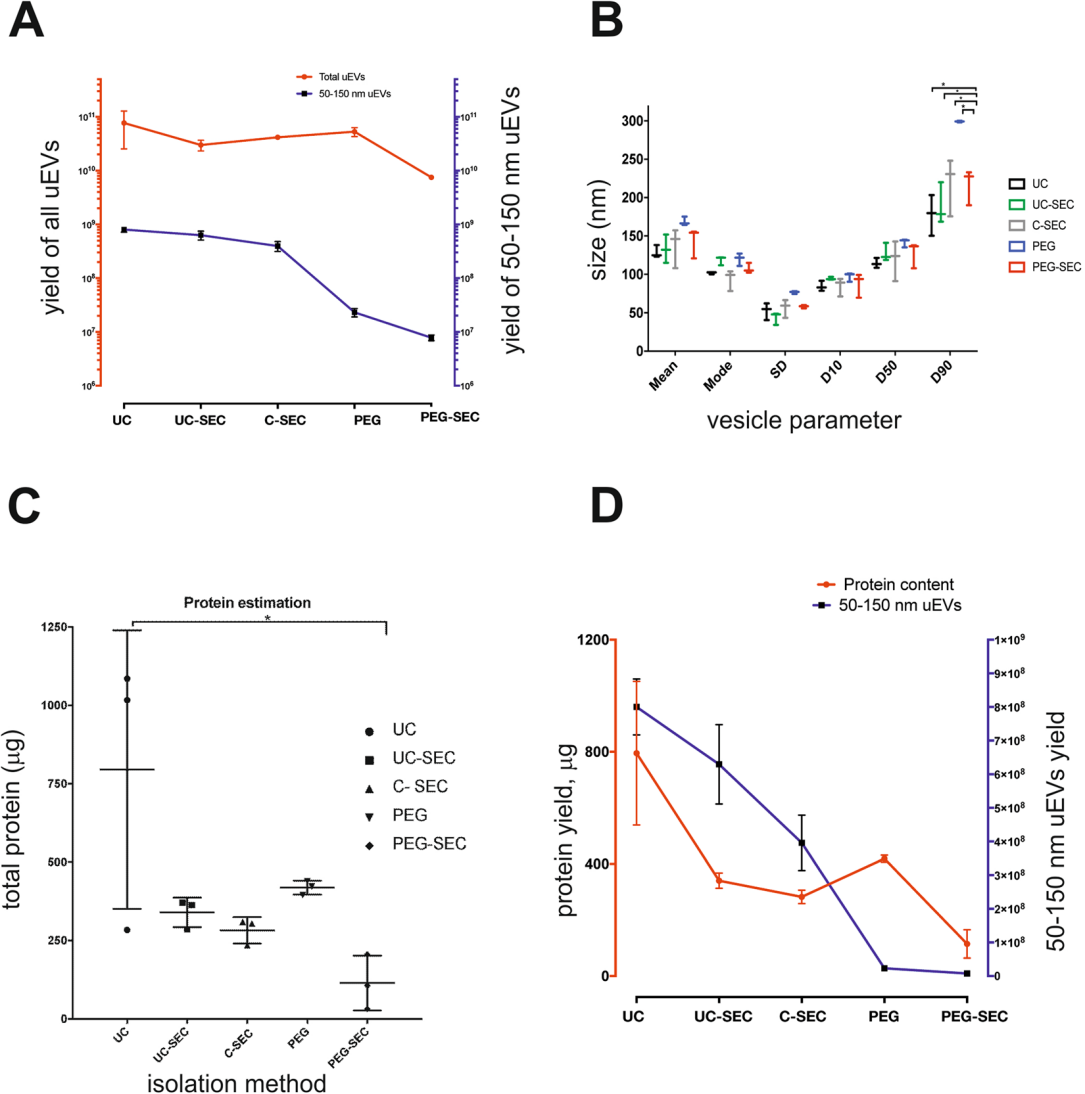
Particle and protein yields of uEVs isolated by different methods. uEVs were isolated from 50 ml of the same urine by 5 different methods: UC = ultracentrifugation, UC-SEC = ultracentrifugation followed by size exclusion chromatography, C-SEC = concentration followed by size exclusion chromatography, PEG = isolation by polyethylene glycol, PEG-SEC = isolation by polyethylene glycol followed by size exclusion chromatography. The results are shown as mean ± SEM of 5 experiments performed in triplicates. (**A**) Number of uEVs isolated by 5 different methods. NTA was performed on isolated uEVs to calculate total particles (left Y-axis in red) and particles in range of 50–150 nm (right Y-axis in blue) in the same sample. (**B**) Size characteristics of uEVs. Mean, mode, standard deviation and different size distributions (D10, D50 and D90) were calculated in samples isolated by 5 different methods. D10 = 10% of particles are below the size indicated as D10, D50 = 50% of particles are below the size indicated as D50, D90 = 90% of particles are below the size indicated as D90. Tukey’s multiple comparisons test showed the most significant differences in D90 between PEG-SEC and all other methods (adjusted p value < 0.1–0.0001). (**C**) Protein content of uEVs measured by BCA. Tukey’s multiple comparisons test was performed and adjusted p-value of UC vs UC-SEC was 0.119, for UC vs C-SEC was 0.0707 and for UC vs. PEG-SEC was 0.0150. (**D**) Correlation between the total protein amount (left Y-axis in red) and 50–150 nm particle count (right Y-axis in blue) of uEVs.

The particles harvested from 50 ml urine using each method were subjected to nanoparticle tracking analysis using NanoSight NS300 as described in Methods. Total particle yield and the content of 50–150 nm uEVs, corresponding to the exosome population were estimated. Ultracentrifugation (UC) yielded higher total uEV count compared to the other tested methods, however, except for PEG-SEC, the difference was not significant due to the high variation between replicates in UC preparations (Fig. 1A). Total uEV count for the PEG method was the second highest after UC, and PEG-SEC yielded the lowest number of uEVs. After gating the uEVs for size (50–150 nm, corresponding to exosomes) and re-analysing the data, the variation which was observed in total uEV count by UC method was significantly lowered indicating that 50–150 nm exosome subpopulation in different replicates of UC was consistent between preparations. Although PEG showed a higher particle yield compared to UC-SEC and C-SEC, after gating for the exosome size, the proportion of 50–150 nm uEVs in these samples was significantly reduced, indicating that PEG precipitated mostly particles within the size range not corresponding to exosomes (Fig. 1A). Tukey’s multiple comparisons test showed no significant difference between the yields of UC and UC-SEC, indicating that there was no significant particle loss during size exclusion chromatography (Supplementary Table ST1).

The size distribution and the average FTLA (finite track length adjustments) of uEVs isolated by different methods were measured by the NanoSight NS300 (Fig. 1B). The mean size of PEG-precipitated uEVs was the highest among all isolation methods but the mode of uEV size was similar across preparations. Size distribution analysis of uEVs isolated with the tested methods showed that PEG-precipitated uEVs contained larger vesicles compared to the other methods, since D90 (90% of particles below the indicated size) of this preparation was significantly higher than D90 of other methods. In contrast, D10 (10% of particles below the indicated size) and D50 (50% of particles below the indicated size) distributions were almost the same in all samples indicating that the isolation method had a negligible effect on the smaller uEVs (Fig. 1B).

In harmony with these results, the uEVs isolated by the UC method show a higher total protein content than other preparations (Fig. 1C). However, due to the variation between UC replicates, the difference was not significant except with the PEG-SEC method. The preparations isolated by PEG precipitation tended to show a higher protein content than UC-SEC, SEC and PEG-SEC, in contrast to the lower number of 50–150 nm uEVs (Fig. 1D). There is a direct correlation between the protein content and the total count of 50–150 nm uEVs in UC, UC-SEC and C-SEC preparations, but no correlation between particle count and total protein recovered by PEG precipitation (Fig. 1D).

### Morphological characterization of uEVs recovered by different isolation methods

**Figure 2:**
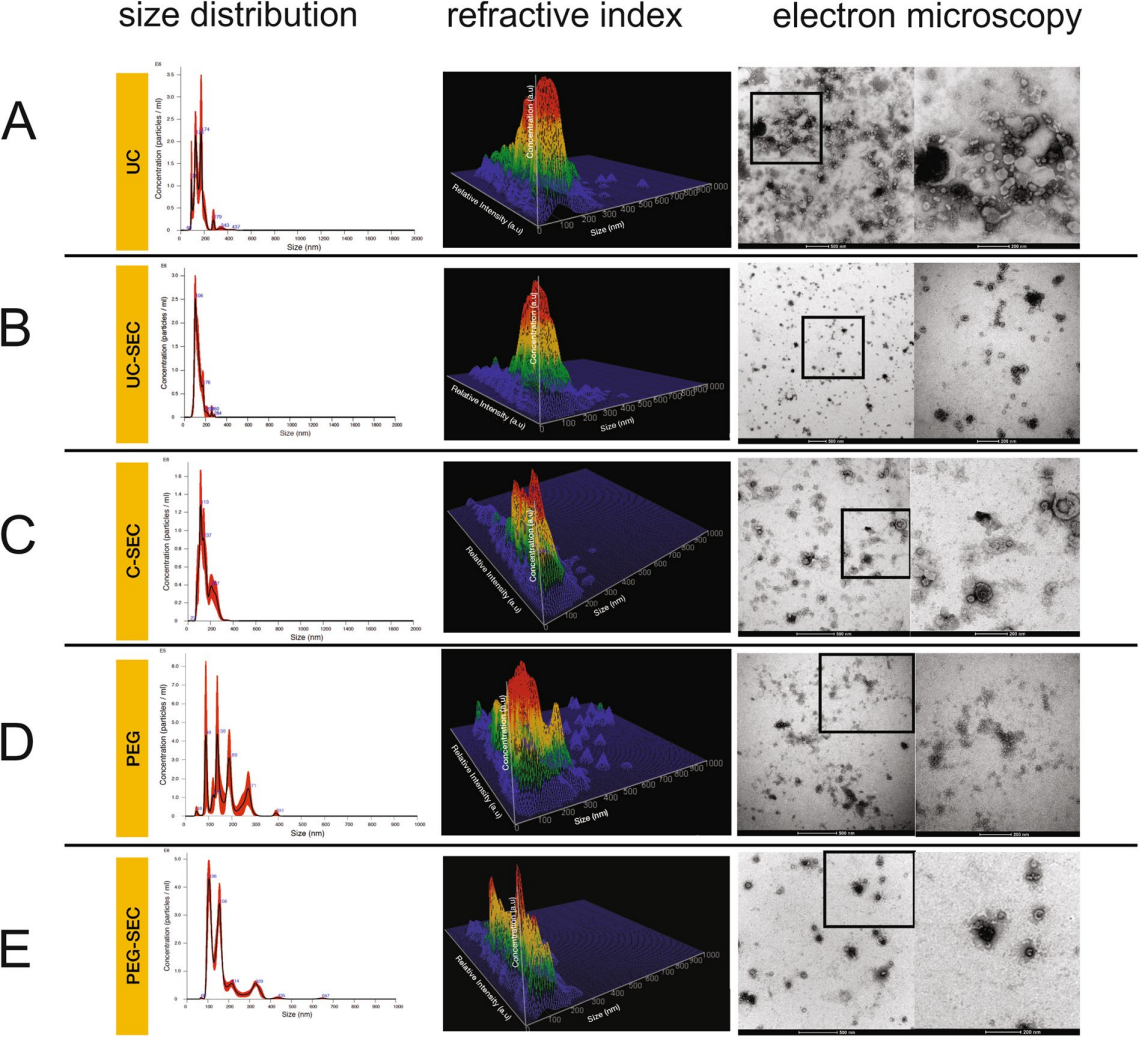
Size distribution, dispersity, and morphology of uEVs isolated by different methods. Size distribution graphs show the concentration on Y-axis and size distribution in X-axis. Refractive index (a.u. = arbitrary units) shows 3D surface plot representing the quantity of light that uEVs scatter in Z-axis, concentration in Y-axis (particles/ml) and size distribution in X-axis. Electron microscopy pictures (images on the left) and a higher magnification of the inset (on the right) show the morphology of particles isolated by each method. Data of representative samples from each isolation method are shown. (**A**) Size distribution of particles, 3D surface graph of refractive index and EM picture (500 nm and 200 nm scale) of uEVs isolated by UC. (**B**) Size distribution of particles, 3D surface graph of refractive index and EM picture (500 nm and 200 nm scale) of uEVs isolated by UC-SEC. (**C**) Size distribution of particles, 3D surface graph of refractive index and EM picture (500 nm and 200 nm scale) of uEVs isolated by C-SEC. (**D**) Size distribution of particles, 3D surface graph of refractive index and EM picture (500 nm and 200 nm scale) of uEVs isolated by PEG. (**E**) Size distribution of particles, 3D surface graph of refractive index and EM picture (500 nm and 200 nm scale) of uEVs isolated by PEG-SEC.

The size and refractive index of uEVs, isolated using five tested methods were determined by Nanoparticle Tracking Analysis (NTA) using NanoSight NS300 as described in Methods, and the particle content and morphology additionally evaluated by negative staining electron microscopy. Introducing the SEC step improved size homogeneity: compare the UC sample (Fig. 2A) with the UC-SEC sample (Fig. 2B). PEG-precipitated uEVs had the highest size heterogeneity (Fig. 2D), although the contaminating high diameter particles were partially removed by SEC in PEG-SEC samples (Fig. 2E). PEG precipitation was not selective for exosome-size range particles, whereas UC-SEC yielded the most homogenous population of vesicles up to 200 nm. In addition to the particle size, NTA records the particle intensity allowing discrimination between particles of a similar size but different refractive index. The refractive index (RI) of exosomes in human urine is reported to be in the order of 1.37^37^. To determine whether the uEV populations isolated using different methods show similar RI, we calculate the RI of isolated uEV using NTA (Fig. 2, refractive index column). uEVs isolated by PEG precipitation showed the most heterogeneous profile containing high and low refractive indices (Fig. 2C), while UC-SEC had mostly low refractive index population in order of 1.37–1.39 (Fig. 2B). UC preparations had low and mid-range refractive uEVs.

To confirm the presence of exosomes and compare the morphology of different uEVs in the samples isolated by different methods, we performed TEM of negatively stained exosomes as described in Methods. EM examination confirmed that vesicles similar in size and morphology to exosomes were isolated using all methods (Fig. 2, electron microscopy column). UC-isolated uEVs were dense and heterogeneous (Fig. 2A), whereas EM pictures of UC-SEC uEVs were more homogeneous and showed cup-shaped vesicles with size range of 50–150 nm (Fig. 2B). Both UC-SEC and C-SEC preparations mostly contained particles while PEG and PEG-SEC preparations were less pure and contained aggregates and other structures (Fig. 2D and E).

Urinary EVs, purified by each method, were used to isolate RNA and subjected to Western blot analysis with antibodies against exosomal marker proteins CD9, CD81, CD63 and TSG101 as described in Methods. Although the total particle count was comparable across all the methods, both PEG and PEG-SEC yielded significantly fewer 50–150 nm particles and RNA, and the total number of exosomes present in these preparations was too low to be detected by Western blotting (Table 1).

**Table 1:** Effect of isolation method on particle count, protein estimation, exosome marker and RNA amount.

Method	Total particle count	50–150 particle count	RNA yield (ng)	Protein estimation (ug)	Detection in WB
UC	9.63E + 07	1.00E + 06	83 ± 5	795.1 ± 45	Yes
UC-SEC	3.63E + 07	7.75E + 05	55 ± 7	340.1 ± 26	Yes
C-SEC	5.88E + 07	4.50E + 05	30 ± 6	282.7 ± 31	Yes
PEG	6.63E + 07	2.88E + 04	3 ± 0.3	418.8 ± 47	No
PEG-SEC	9.38E + 06	9.63E + 03	1 ± 0.4	114.78 ± 11	No

### Optimization of differential ultracentrifugation and size exclusion chromatography method (UC-SEC)

**Figure 3:**
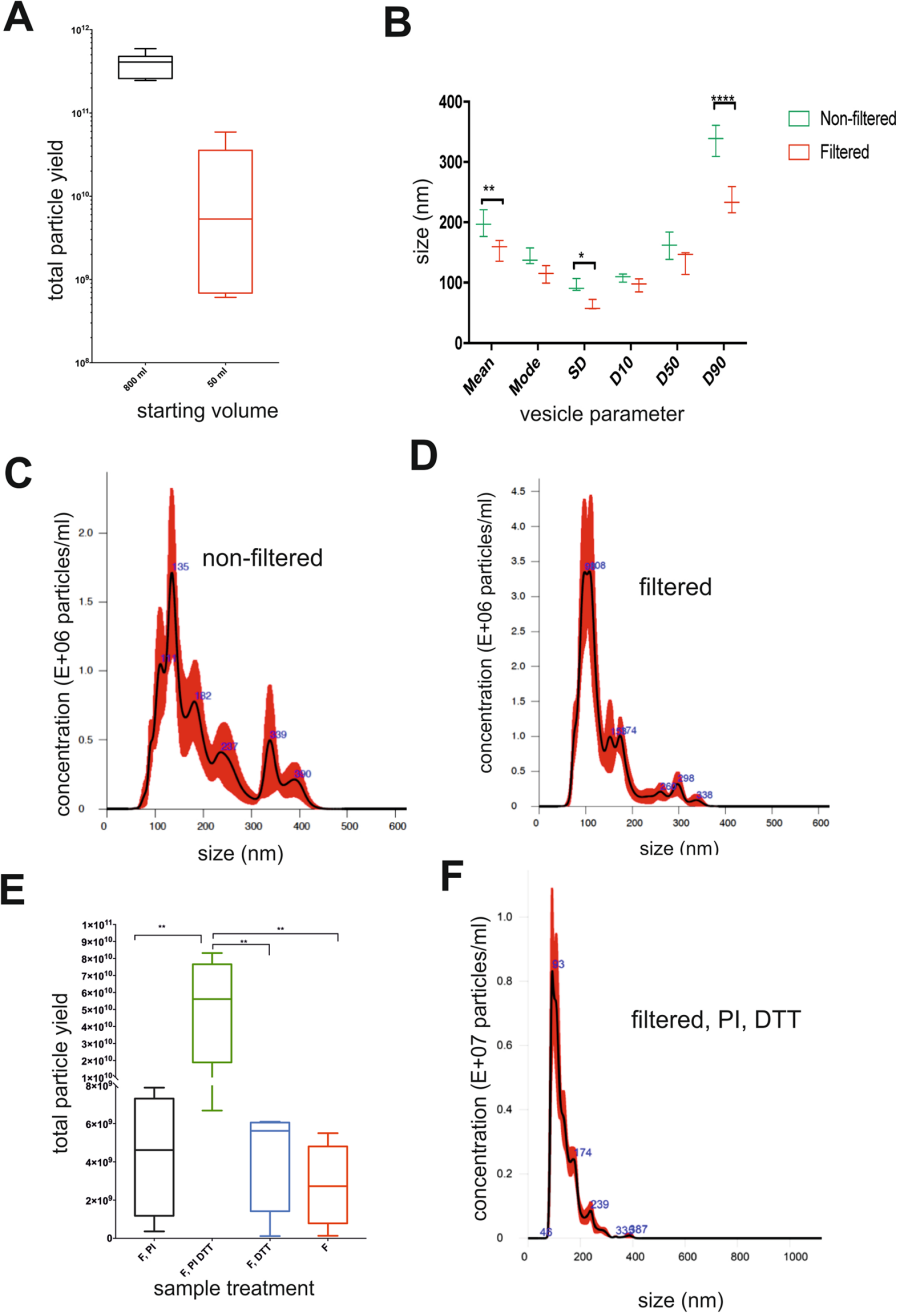
Optimization of the UC-SEC protocol. (**A**) Effect of starting urine volume on the particle count and experimental variation. UC-SEC was done using 800 ml or 50 ml of the same urine sample and total particles counted by NTA. The data are the mean ± SEM of 6 independent experiments. (**B**) Filtration significantly reduced the mean size (adjusted p values < 0.0064), SD indicating variation in sub-populations (adjusted p values < 0.0360) and size of bigger particles (D90) (adjusted p values < 0.001) in filtered urine. (**C**) Size distribution and concentration of particles in a non-filtered urine sample. The most particle-rich SEC fraction from a representative experiment is shown. (**D**) Size distribution and concentration of particles in a filtered urine sample. Urine filtration results in a more homogenous particle size distribution compared to non-filtered samples. The most particle-rich SEC fraction from a representative experiment is shown. (**E**) Combination of filtration, PI and DTT treatment resulted in highest yield of uEVs isolated using SEC. All urine samples were filters using 0.22 um filters. The data are the mean ± SEM of 4 replicates, adjusted p-value <0.05. (**F**) Combination of filtration, PI and DTT treatment on size distribution of particles. The most particle-rich SEC fraction from a representative experiment is shown.

Based on the quantity, size and morphology of uEVs we selected UC-SEC as the best suitable for urinary exosome isolation and further optimized it to achieve the maximum yields of particles with the desired size-range (50–150 nm) and purity. We tested whether the UC-SEC method is applicable when using higher urine volumes by using a significantly different starting volume of urine (800 ml vs. 50 ml). The total particle count was increased depending on the starting urine volume (Fig. 3A), indicating that at this range the Sepharose SEC column was not saturated. Loading a more concentrated UC sample from the 800 ml preparations consistently increased the particle yield after SEC separation. With the same capacity factor (the degree of retention of a solute (exosomes) relative to an unretained peak), loading a more concentrated sample results in a non-linear increase of eluted exosomes proportional to the retained particles. This gives a higher concentration of the eluted fractions in 800 ml sample vs. 50 ml sample. When possible, increasing the starting urine volume would be advisable as these preparations showed lower variation of final particle yields (Fig. 3A).

Filtering processed urine through a 0.22 μm filter immediately before ultracentrifugation had an effect on the particle size distribution: the filtered samples had significantly fewer sub-populations (smaller variation) and lower average size compared to non-filtered urine (Fig. 3B). This particularly affected the larger particles (Fig. 3B, D90 values), present in non-filtered samples (Fig. 3C) and removed by filtration (Fig. 3D). The total particle number in filtered and non-filtered samples was similar, indicating that filtration did not affect the yield of exosomes (data not shown).

**Figure 4:**
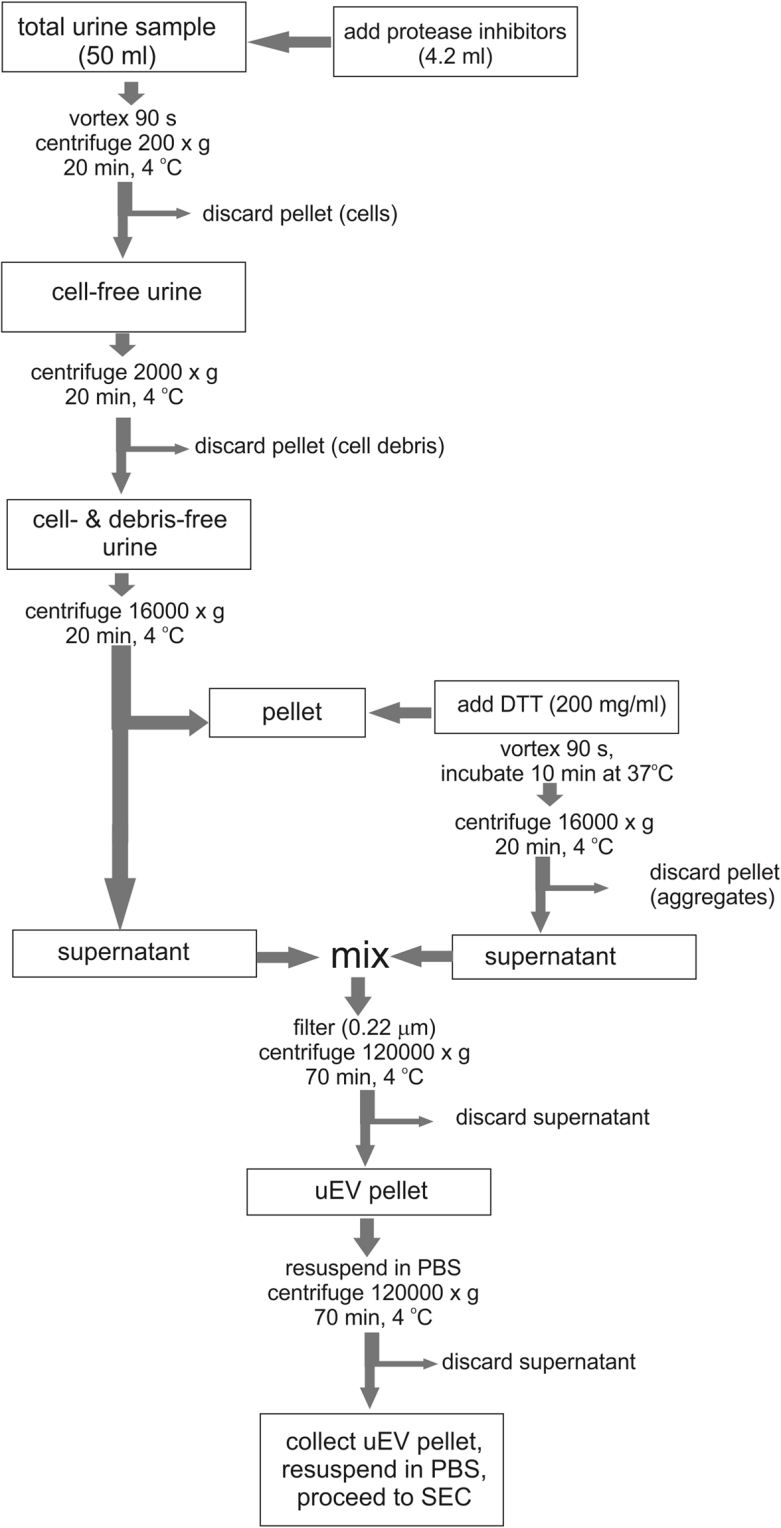
The optimized UC-SEC method overview Midstream, clean catch first-morning urine was collected. Urine samples (50 ml) were mixed with 4.2 ml of protease inhibitor (PI) solution immediately after urine collection. Urine was vortexed and centrifuged at 200 × g for 20 min to remove cells. After discarding the pellet, the cell-free urine was centrifuged at 2000 × g for 20 min to remove cell debris and large protein aggregates. Supernatant was centrifuged at 16000 × g for 20 min to remove ectosomes and other large particles. Supernatant was collected and kept in 4 °C. The pellet was treated with DTT (200 mg/ml) and incubated 10 min in 37 °C. After a short vortex the mixture was centrifuged at 16000 × g for 20 min and supernatant was pooled with the supernatant from last 16000 × g centrifugation step. The pooled supernatants were filtered through 0.22 μm filters and ultracentrifuged at 120000 × g for 70 min. The supernatant was carefully decanted, the pellet overlaid with particle-free PBS and subjected to ultracentrifugation at 120000 × g for 70 min. The final pellet was resuspended in 500 μl of particle-free PBS. The resuspended pellet was used for SEC, NTA, protein quantification and morphology evaluation by EM.

Treating filtered urine with protease inhibitors (PI) and DTT before subjecting it to UC-SEC significantly increased the yield of total particles (Fig. 3E). NTA analysis of this preparation showed high size homogeneity within the range of 50–200 nm (Fig. 3F), indicating that most uEVs were exosomes. The optimized UC-SEC protocol which was used in all subsequent applications is shown in Fig. 4.

### Characterization of protein markers and RNA load of exosomes, purified using the UC-SEC method

**Figure 5:**
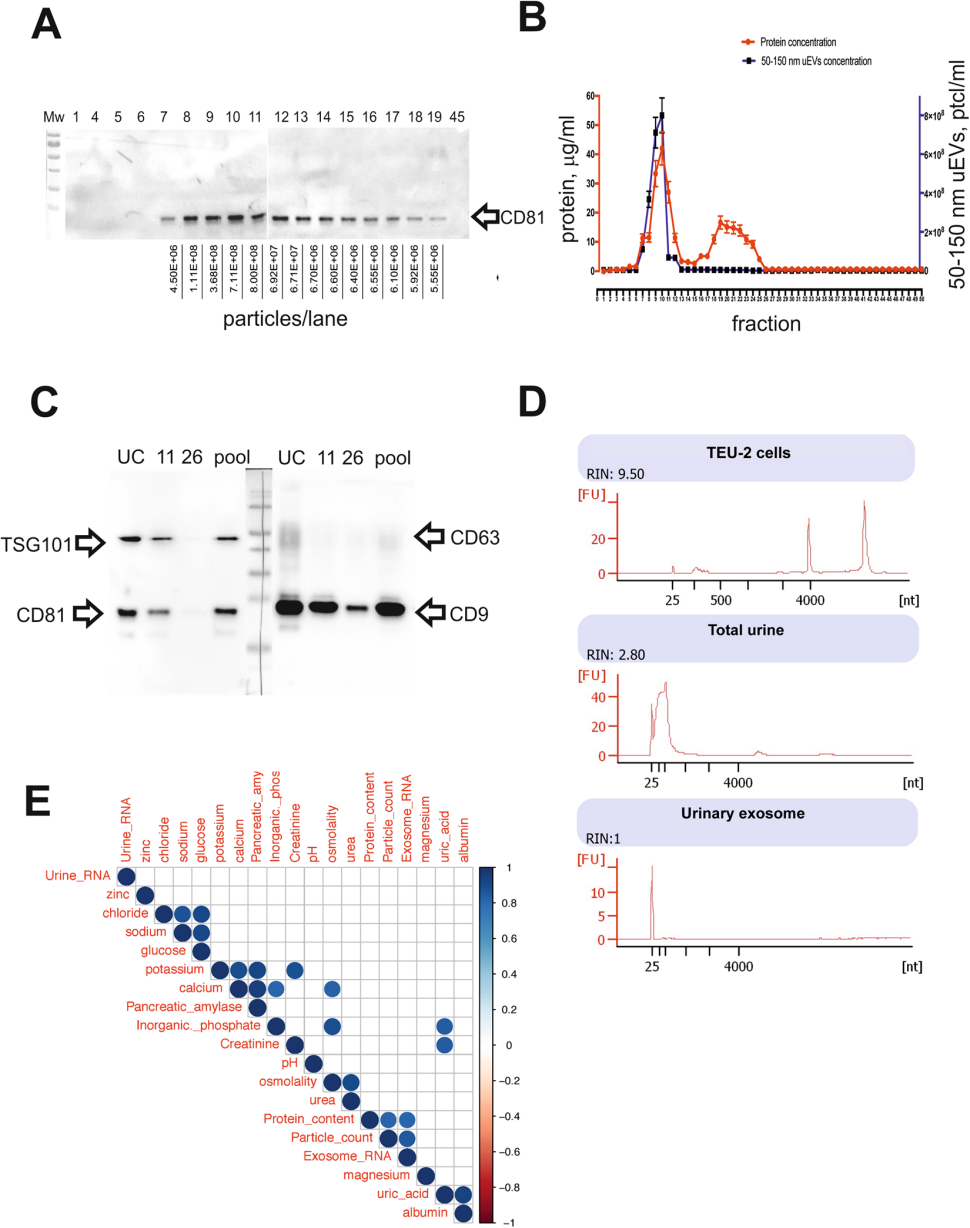
Protein markers and RNA load of exosomes, purified using UC-SEC. (**A**) In a representative experiment, 20 μl of fractions 1, 4–19 and 45 were tested by Western blotting with anti-CD81 antibody. NTA was used to count 50–150 nm particles in all SEC fractions, and the particle content of each lane indicated. Uncropped images of 2 blots (9 samples each + Mw markers) are presented. (**B**) Particle and protein concentrations in 50 fractions of the UC-SEC. The data are the mean ± SEM of 3 independent experiments. (**C**) Exosomal markers CD9, CD63, CD81 and TSG101 were tested by Western blotting. Shown are the UC pellet subsequently used for SEC, fractions 11 and 26 of the SEC and a pool of all exosome-containing SEC fractions of a representative experiment. One membrane was cut and probed with anti-TSG101 + anti-CD81 (left) or anti-CD63 and anti-CD9 (right), and uncropped images combined. (**D**) Total RNA isolated from TEU-2 cells, total urine and urinary exosomes from UC-SEC was analysed by NanoChip. Length of detected RNA species is indicated (nt). (**E**) Pearson correlation between urine contents (chemical composition and total RNA) and uEV parameters (exosome miRNA read count, protein content and particle number). Only Pearson correlations with p-value ≤ 0.05 are shown (blue for positive correlation), the intensity of blue colour corresponding to the degree of correlation. The data are diagnostic values for urine composition and RNA, protein and exosome concentrations of 6 total urine samples, processed by the optimized UC-SEC method.

To further characterize the uEVs, isolated using from 50 ml of urine using UC-SEC, we analysed each fraction collected from SEC by Western Blotting with anti-CD81 antibodies. In a representative experiment, CD81 was detected between fractions 7 to 19, with the peak antibody binding in fractions 10 and 11, in good agreement with NTA particle count (Fig. 5A, particles loaded on each lane are shown below). Comparison of the particle numbers and protein content in fractions of 3 UC-SEC preparations (Fig. 5B) indicated a good correlation in fractions 8 to 12, with some non-particulate protein contaminant eluted in fractions 17 to 25 (Fig. 5B). Overall, the fractions containing the highest protein concentration also had the highest particle numbers in size range of 50–150 nm. Protein concentration in the peak SEC fraction ranged between 40–90 μg/ml, depending on the detection method (Supplementary Fig. S3). The minimum of 5.5E + 06 particles per lane (size range 50–150 nm) were detectable with CD81 antibody. We evaluated the presence of the other exosomal markers including CD9, CD63, TSG101 and CD81 in the UC-SEC preparation (Fig. 5C). All markers were detected in the UC-isolated uEVs, in fraction 11 of UC-SEC (highest particle concentration by NTA), and in the pool of all particle-containing fractions (10–20). CD9 was positive in fraction 26 (lowest particle concentration by NTA), indicating a high sensitivity of the antibody and high levels of CD9 in uEVs (Fig. 5C).

We purified RNA from total urine and urinary exosomes isolated by UC-SEC and compared the RNA species profiles with the total RNA isolated from TEU-2 immortalized human ureteral cell line^38^. Unlike cellular RNAs, which included both small and long species, mostly small RNAs were detected in both total urine and urinary exosomal RNA preparations (Fig. 5D). In contrast to the total urine RNA, which showed a higher size variation and included long RNAs (over 4000 nt), the urinary exosome RNAs were predominantly 25 nt long (Fig. 5D).

In six independent preparations, we sought to find a correlation between the chemical composition of urine and the urinary particle parameters such as particle counts, RNA content and protein content of urinary exosomes. We performed a full urinalysis for the total urine samples which were subjected to UC-SEC followed by NTA, protein estimation and RNA isolation (Supplementary Table ST2). All the samples had normal parameters (bilirubin, urobilinogen, ketones, ascorbic acid, glucose, protein, blood (hemoglobin, erythrocytes), nitrite, leukocytes, pH and density as determined by the diagnostic lab of Inselspital, Bern, Switzerland (Supplementary Table ST3). We used corrplot package in R to find the correlation between the chemical composition of urine, NTA results, protein quantification and RNA analysis (Fig. 5E for the significant correlations). There was no correlation between the particle parameters and the chemical composition of urine samples, but a significant correlation between particle count, exosome RNA content and protein quantification of exosome samples. Creatinine, widely used as a normalizer of urinary content, showed a negative correlation with urinary RNA, though this did not reach significance (Supplementary Fig. S4 for all correlations). Interestingly, total urine RNA did not correlate with exosome RNA, which might be due to the high incidence of free, non-exosomal species.

### miRNA profiling in urinary exosomes and total urine

**Figure 6:**
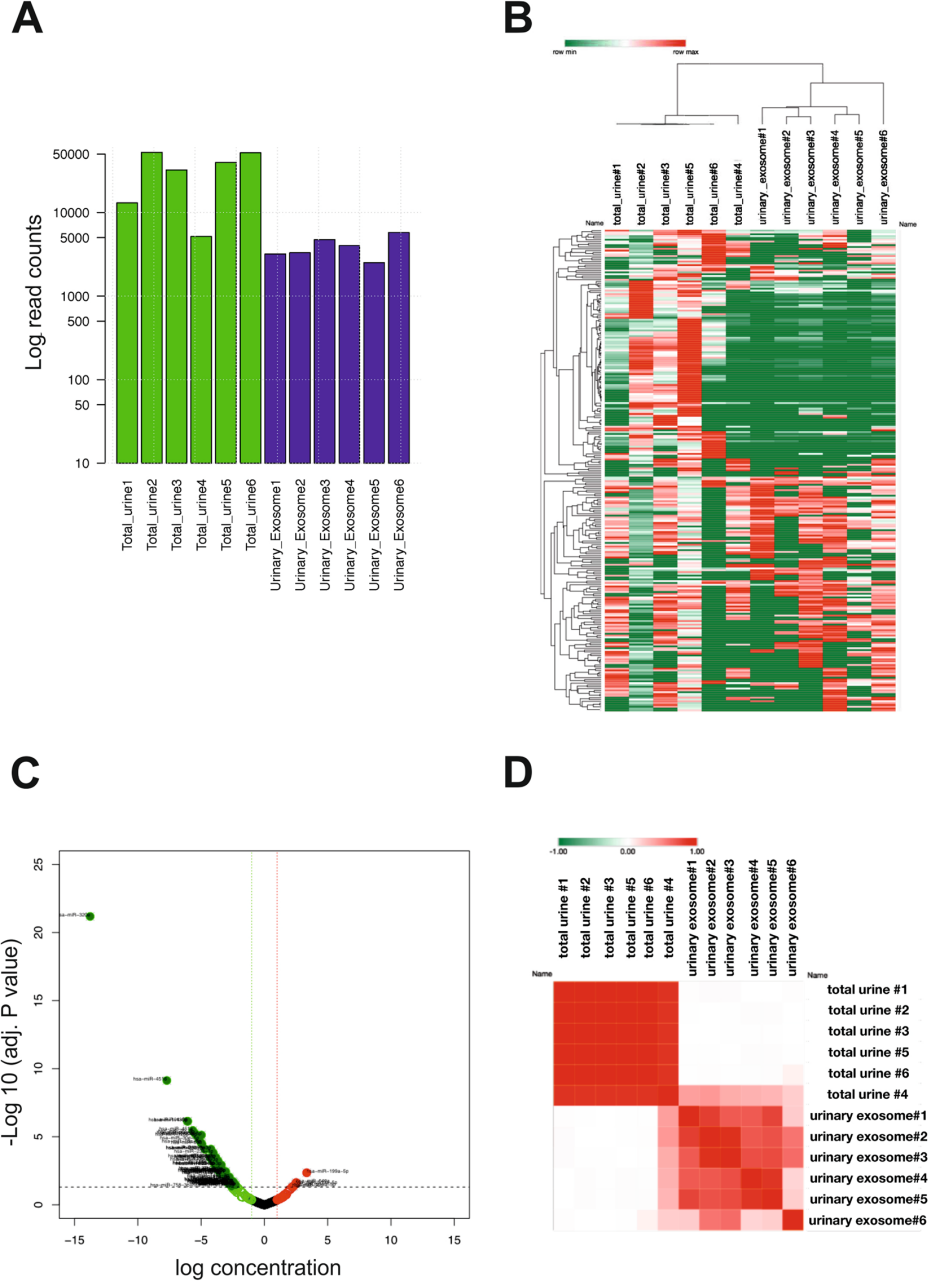
miRNA profiling in the total urine and uEVs. RNA samples were derived directly from 50 ml of total urine or from uEVs, isolated by UC-SEC from 50 ml of the same urine sample, and miRNAs were profiled using NanoString. (**A**) Bar chart representing the read count differences between different samples. Read counts are shown in logarithmic scale. The data are sum of all detected miRNA (normalised read counts of 6 paired experiments). (**B**) Hierarchical clustering and heatmap of 256 miRNAs expressed in the total urine and urinary exosomes isolated from the same sample. Each row represents one miRNA and each column represents one sample. Expression levels are colour-coded (bar above, green for low and red for high levels). (**C**) Volcano graph showing the log10 fold difference of miRNA content in urinary exosomes compared to total urine. The black dashed line represents the p-value of 0.05. Green dash line shows −1.5 fold lower and red dash line shows 1.5 fold higher content of miRNAs in exosomes compared to total urine. (**D**) Similarity matrix for total urine and urinary exosome samples. The redder a field is, the more similar the samples are in terms of miRNA expression.

We established that the RNA load of 50–150 nm uEVs purified by UC-SEC contained mostly 20–25 nt RNA species, corresponding to the miRNA size (Fig. 5D). Therefore, we sought to identify the miRNAs packaged into the exosome vesicles and compare it with the miRNA profile of the original urine samples. Urinary exosomes were purified from 50 ml urine by UC-SEC, and RNA isolated from uEVs-containing SEC fractions. In parallel, total RNA was isolated from 50 ml of the same urine sample. NanoString nCounter was used to profile 800 human miRNAs. The miRNA read counts in the total urine were higher than in the exosomal RNA preparations from the same urine samples (Fig. 6A), indicating that not all RNA is packaged. We could stably detect 256 miRNAs in all total urine preparations (Supplementary file Nanostring data.xls) and used these to investigate the hierarchical clustering of the samples. Total urine samples were clustered together whereas urinary exosomes fall into a separate cluster (Fig. 6B). Volcano graph (Fig. 6C) shows the log2 fold difference of urinary exosome miRNAs compared to total urine miRNAs. The exosome contents of the majority of miRNAs is lower than their amounts in the total urine. Because each total urine RNA sample had its corresponding exosome RNA sample, we calculated the Euclidean distance of samples and presented it as a similarity matrix (Fig. 6D). Similar to the heatmap (Fig. 6B), the results clustered in two groups: total urinary RNA group and exosomal RNA group (Fig. 6D).

### Packaged and naked miRNAs identified in uEVs and total urine

**Figure 7:**
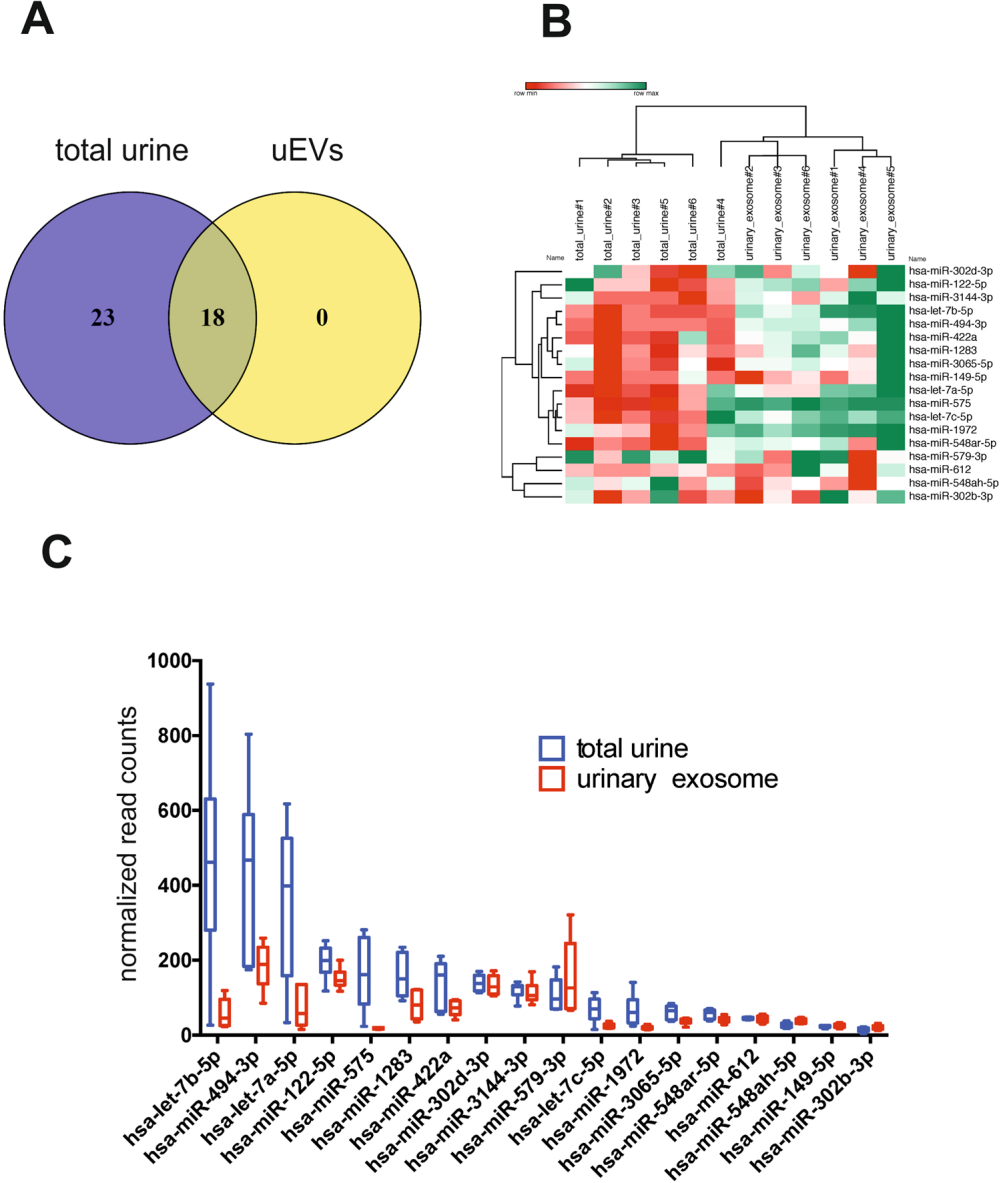
miRNAs present in both the total urine and urinary exosome samples. (**A**) Venn diagram showing miRNAs stably detected all 6 sample pairs of total urine and urinary exosomes. All 18 miRNAs detected in uEVs are also present in the starting total urine samples. (**B**) PCR validation for NanoString data of 18 intersecting miRNAs. Heatmap and clustering of shared miRNAs indicate their lower levels in uEVs. (**C**) Expression levels of 18 common miRNAs detected by NanoString. The high and low abundance of miRNA correlated in total urine samples and urinary exosomes. The differences were statistically significant (p ≤ 0.05).

When we narrowed down the inclusion criteria by only considering miRNAs, detected in all 6 total urine samples, and in all 6 uEVs samples, 41 miRNAs were detected in the total urine while 18 were detected in the exosomes (supplementary file Nanostring data.xls). The 18 exosomal miRNAs were also detected in the total urine (Fig. 7A), indicating that no miRNAs were present exclusively in exosomes. When we interrogated the dataset for the miRNAs, present in total urine samples and not detected in any of the uEV samples, we identified 15 miRNAs only present in the total urine (Table 2). We validated the NanoString results using qPCR and detected all 18 shared miRNAs in both total urine and urinary exosome samples (Fig. 7B). Similar to the NanoString data, the hierarchical clustering of samples based on the levels of these 18 miRNAs shows two groups, total urine and urinary exosomes. This is due to the generally lower levels of these miRNAs in the exosome samples (Fig. 7C). These results are not surprising, taking into account the material losses at various steps of US-SEC purification. Our data also indicate that there is no obvious enrichment of miRNAs in uEVs as none of the 18 miRNAs were exclusively present or detected significantly higher in exosomes than in the total urine samples (Fig. 7C). The miRNAs, shared in total urine and packaged in uEVs, and those only detected in the total urinary RNA preparations and not in the uEVs are listed in Table 2.

**Table 2:** NanoString read counts of exosomal and naked miRNAs.

Name	Avg urine	Avg exosome
**Shared miRNAs**		
hsa-let-7b-5p	463.3	58.4
hsa-miR-494–3p	436.0	184.4
hsa-let-7a-5p	357.1	72.7
hsa-miR-122–5p	196.3	151.4
hsa-miR-575	164.1	18.2
hsa-miR-1283	158.8	81.1
hsa-miR-422a	139.4	72.3
hsa-miR-302d-3p	139.0	133.5
hsa-miR-3144–3p	120.7	113.9
hsa-miR-579–3p	108.3	155.8
hsa-let-7c-5p	68.8	25.5
hsa-miR-1972	66.7	20.4
hsa-miR-3065–5p	61.4	38.1
hsa-miR-548ar-5p	56.6	41.9
hsa-miR-612	44.8	43.6
hsa-miR-548ah-5p	27.7	37.7
hsa-miR-149–5p	23.4	25.2
hsa-miR-302b-3p	15.0	20.7
**Urine-exclusive miRNAs**		
hsa-miR-320e	22217.1	1.0
hsa-miR-4516	328.2	1.0
hsa-miR-451a	92.8	1.0
hsa-miR-1305	103.0	1.0
hsa-miR-29c-3p	78.4	1.0
hsa-miR-10a-5p	57.0	1.0
hsa-miR-23b-3p	46.6	1.0
hsa-miR-30e-5p	62.9	1.0
hsa-miR-26b-5p	47.3	1.0
hsa-miR-194–5p	132.0	1.0
hsa-miR-223–3p	25.9	1.0
hsa-miR-22–3p	76.9	1.0
hsa-miR-1285–5p	69.0	1.0
hsa-miR-630	46.1	1.0
hsa-miR-148b-3p	26.6	1.0

## Discussion

In this study, different urinary exosome isolation methods were compared in a systematic manner to define the most appropriate isolation procedure suitable for biomarker discovery, exosome transfer experiments and any other downstream procedure, requiring a highly pure urinary exosome sample. The existing methods of urinary exosome isolation are based on ultracentrifugation or polymer co-precipitation, the latter is often used in commercial kits, and suffer from insufficient yield, low purity and lack of consistency^39^. In particular, the experiments involving exosome-mediated transfer of cargo require high sample purity, which should guarantee the selectivity and specificity of the observed effects^40^. The modified UC-SEC method presented in this study utilizes protease-protected and concentrated urine (either by ultracentrifugation or using concentrator columns) followed by size exclusion chromatography, and results in high numbers of pure and size-homogeneous uEVs. UC-SEC purified uEVs are free from contaminating soluble urinary proteins and DTT, allowing the downstream analysis of their cargo and functional assays.

Although the UC method produces in the highest particle yield, this difference is not significant compared to other methods due to a high variation. This variation was reported in other studies comparing commercial kits with the UC method^41^ and can originate from steps such as decanting the supernatant or resuspension of the pellet. Our results show that uEVs isolated by UC method have high dispersion and contain protein aggregates and other particles, visible in EM pictures. The UC samples showed the highest mean and variation of protein concentration compared to the other methods, indicative of protein contamination. After SEC, the protein content of exosome fractions was proportional to the particle number and the contaminating proteins were in the non-exosomal fractions. Our results also suggest the presence of potential vesicle aggregates in the UC pellet, which is reflected in NanoSight graphs.

We used fixed-angle rotors as they provide a lower k-factor and a better separation than swinging-bucket rotors. Fixed-angle rotors produce a less condensed pellet compared to swing buckets, contributing to the observed variability of the total particle count in the UC preparations. A recent study demonstrated that viscosity has a significant correlation with the recovery of EVs^42^, further contributing to the differences in the uEV yield because of the difference in starting urine viscosity. Moreover, UC may cause vesicle rupture, causing vesicle loss during the ultracentrifugation process (splat factor). UC-SEC had a significantly lower yield of total particles compared to the UC method. However, the difference in particles count for 50–150 nm uEVs was not significant. EM pictures of the UC-SEC preparations show uEVs in the range of 50–200 nm, less non-cup-shaped vesicles and fewer vesicle aggregates in the UC-SEC isolated samples. The protein amounts in the exosome fractions of UC-SEC was in good agreement with the particle count determined by NTA, indicating high purity of the resulting vesicles. PEG and PEG-SEC preparations did not show exosomal marker bands in the Western blot and contained significantly less 50–150 nm particles in EM pictures. We conclude that although PEG-based methods may potentially isolate particles, the particle count is not high enough for these samples to be detected by Western blotting or these particles are not exosomes. The size distribution, average FTLA and the reflective index of all methods except PEG and PEG-SEC were not significantly different. PEG and PEG-SEC produced several populations bigger than 200 nm suggesting clumping of particles during the isolation process or presence of bigger non-exosome vesicles. Altogether, our data suggest that starting from the same urine volume (50 ml), UC-SEC is the best method yielding high purity exosomes in a concentration allowing their detection in SEC fractions by Western blotting with anti-CD81 antibodies. NTA analysis shows that urinary vesicle populations are polydisperse, therefore for a clean transfer experiment, SEC is beneficial since it can fractionate the vesicles by their size.

We further improved the yield and purity of the UC-SEC-isolated uEVs by including filtration, protease inhibition and THP solubilisation by DTT treatment. Filtration effectively removed the sub-population of vesicles larger than 220 nm, enriching the resulting uEVs in exosomes. A combination of protease inhibitors and DTT significantly increased the uEVs yield. DTT reduces the disulfide bonds of proteins and peptides^43^, giving raise to concerns of its use due to interference with the biological activity of the exosomal surface proteins, such as sortilin-related receptor and megalin, especially in the downstream cargo transfer assays^44^. Therefore, THP removal from urine by high salt precipitation was suggested as an alternative^43^. The reducing effects of DTT are reversible and disulfide bonds begin forming as soon as the reducing agent is removed^45,46^, giving SEC an additional advantage as it effectively purifies exosomes from DTT. We were able to detect DTT residuals in fractions 28–35 of SEC, which were apart from exosome-containing fractions (7–19) and did not contain any particles on NTA.

Using six sample pairs (total urinary miRNAs vs. uEV-derived miRNAs from the same starting material) we isolated and characterized the RNA load of the uEVs and compared it with the RNA present in total, unfractionated urine. Both types of RNAs were enriched in small RNA species, although the uEV-packaged RNAs showed less size variation and was composed of 20–25 nt RNAs, consistent with miRNA size. Quantification of miRNAs present in total urine and miRNA content of uEVs revealed a significant difference in read counts: up to 50 times more miRNA reads were detected in the total urine compared to the exosome miRNAs isolated pair-wise using the same input urine. We show that from 41 miRNAs robustly detected in total urine, 18 were also detected in urinary exosomes. The 23 remaining miRNAs were detected in all 6 total urine samples, but only in 5 or less out of 6 exosomal samples. We also found 15 naked miRNAs including miRNAs hsa-miR-320e, hsa-miR-4516, hsa-miR-451a and hsa-miR-194–5p, which were not detected in any of the exosomal RNA preparations, but present in at least 5 out of 6 total urinary RNAs. Overall, the miRNAs detected in exosomes represented about 43% of all miRNAs expressed in total urine suggesting exosomes are major carriers of miRNAs in urine. The amounts of exosomal miRNAs were lower than the amounts of total miRNAs from the same samples, indicative of them being a sub-population. For this reason, some of low abundant urinary circulating miRNAs might not be detected in exosomes. On the other hand, the expression levels of naked miRNAs hsa-miR-320e and hsa-miR-4516 were particularly high in the unfractionated urine, indicating that their absence in exosomes was not due to the detection failure. Such miRNAs might indeed be excluded from uEVs and processed into the urine by direct filtration or other mechanisms.

A very variable miRNA content of urinary exosomes has been reported^47^. Ultracentrifugation is the most often used exosome isolation method in these studies, which is inconsistent and prone to contamination with other, non-exosomal protein-RNA complexes. Unfortunately, there is no consensus of standard protocols for urinary exosomes isolation and often small technical modifications have a considerable impact on the final results^48^. The approach presented here is highly reproducible and offers a good correlation between the protein and RNA yields of the uEVs with the particle number counted and characterized by NTA and EM. On the other hand, our results demonstrate that using urinary components such as creatinine for normalization leads to bias in result interpretation, as they do not correlate with the exosomal cargo. The optimized UC-SEC procedure, proposed in this study (Summarized in Fig. 4) is suitable for uEVs purification and isolation of exosomal miRNA for biomarker discovery.

## Methods

### Study approval

Permission to conduct this study was obtained from the local Ethics Committee (KEK 2017–01609), and all subjects gave written informed consent. The methods in this study were carried out in accordance with the approved guidelines by University of Bern and University Hospital of Bern and all experimental protocols were approved by the ethics committee.

### Sample collection and processing

All first-void mid-stream urine samples were collected from healthy male volunteers (age range between 20–55 years old) directly into sterile 50 ml plastic centrifuge tubes (Starstedt). These samples were then pooled for downstream procedures. To normalize the procedure for different isolation methods, a general approach was followed (Supplementary Fig. S1). First-morning urine was used for an enrichment of exosomes in the total urine overnight. Processing by serial centrifugation was used to purify the total urine: first centrifugation at 200 g for 20 min (Eppendorf, Centrifuge 5804 R) to remove dead and alive cells, then at 2,000 g for 20 min (Eppendorf, Centrifuge 5804 R) to remove cell debris, bacteria and other proteins. Finally, urine was depleted of ectosomes at 16,000 g for 20 min (Kontron, fixed angle rotor TFT70.38, TGA-65 ultracentrifuge). All centrifugations were done at 4 °C. Clearing factors were used to calculate the right number of G for the centrifugation and centrifugation time were verified prior to this study^49^. The processed urine sample pool was divided and 50 ml fractions used in every isolation method, repeated at least 3 times.

### Depletion of Tamm-Horsfall Protein (THP), filtration and protease inhibition

THP was removed by incubating the pellets after 16,000 g centrifugation step in DL-dithiothreitol (DTT) (Sigma), final concentration 200 mg/ml at 37 °C for 10 min^50^. During the DTT or vehicle incubation, samples were vortexed every 2 min. This procedure denatures the zona pellucida domains in the THP, solubilizing and allowing it to be removed in the supernatant at the subsequent centrifugation. Urine was filtered after DTT treatment using 0.22 µm filters (Merck & Millipore, Millex-GP Filter Unit, cat no. SLGP033RS). 4.2 ml protease inhibitor (PI) mix [10.855 mg NaN3, 6.875 mg 4-(2-Aminoethyl)benzenesulfonyl fluoride hydrochloride (AEBSF), 50 µL Leupeptin (1 mg/ml in H_2_O) and 4.17 ml in H_2_O] was added to each 50 ml urine immediately after sample collection^51^.

### Concentration of urine sample

Urine samples were clarified by vacuum filtration using a Steriflip® filter unit [#SCGP00525; 0.22 μm Millipore Express PLUS (PES) membrane]. Amicon® Ultra-15 filter (#UFC901024,10 kDa MWCO) was Equilibrated using PBS with centrifugation at 4000 g for 10 min. 15 ml of urine was added to the device and centrifuged at 4000 g for 30 min to concentrate. The procedure was repeated until the whole urine sample (50 ml) was loaded.

### Ultracentrifugation (UC)

The processed urine samples were centrifuged at 120,000 × g for 70 min at 4 °C (Beckman Coulter, Rotor Type 45 Ti, Optima L-90K Ultracentrifuge). The fixed angle rotor was chosen for better pelleting efficiency due to its lower K- factor. The supernatant was discarded and the pellet diluted in 25 ml particle free PBS (Gibco, cat no. 10010023, pH7.4) and centrifuged at 120,000 × g for 70 min at 4 °C. The supernatant was discarded and the pellet containing the exosomes was resuspended in 500 µl of particle-free PBS.

### Isolation of uEVs using polyethylene glycol (PEG)

A polyethylene glycol-based uEV isolation solution was prepared by mixing 100 g PEG (average molecular weight of 8,500–11,500, Sigma, Cat No. 309028) and 6 g NaCl and combined with 250 ml sterile-filtered H_2_O (18.3 Mega-ohm system; ELGA Purelab flex purification system). The PEG stock solution was analysed by nanoparticle tracking (the NanoSight NS300 instrument) to confirm it contains no particles. One part of the PEG stock solution was added to 4 parts of the urine (v/v), the samples were thoroughly mixed, incubated at 4 °C at least 12 h (overnight) and centrifuged at 1000 g for 30 min at 4 °C (Eppendorf, Centrifuge 5804 R). The excess PEG was aspirated and the resulting pellet resuspended in 500  μl of particle-free PBS (pH 7.4). For some samples, the PEG-precipitated pellet was resuspended, ultracentrifuged at 100,000 g for 70 min to wash the particles of contaminating protein and PEG. All samples were finally resuspended in 500 μl particle-free PBS.

### Size exclusion chromatography (SEC)

A peristaltic pump (Type MS-4 Reglo B, Ismatec) was used to pack the chromatography column (Biorad, Econo-Column® Chromatography Columns, 0.7 × 20 cm #7374721) with 2% cross-linked agarose gel filtration media (Sepharose CL-2B, GE Healthcare, 17014001, sepharose height = 13 cm) (Supplementary Fig. S2). A porous frit was positioned at the top of the Sepharose to avoid its disturbance during sample loading and elution. The column was washed twice with citrate buffer (0.32% citrate in PBS, filtered through 0.22 µm filter). The optimum volumetric flow rate (VFR) was calculated using the following formula:

Volumetric flow rate (ml/min) = (recommended linear flow rate LFR (cm/h)/60) × column cross sectional area (cm2), where LFR = 30 cm/h. Diameter (d) = 0.7 cm. Volumetric flow rate (ml/min) = (30/60) × ((p × 0.72)/4) = 2.44 ml/min

500 μl of sample, either urine after concentration (C-SEC), or resuspended pellets after PEG precipitation (PEG-SEC) or ultracentrifugation (UC-SEC), was loaded onto the column and fifty 0.5 ml fractions corresponding to the void volume peak were collected. The pump was either pulling or pushing with a constant flow rate. After chromatography, the column was washed with 1M NaCl and stored at 4 °C for further use.

### Estimation of protein concentration

Protein concentration was assessed by the Bradford (Bio-Rad Laboratories) or Bicinchoninic acid (Pierce™ BCA Protein Assay Kit, Catalogue number: 23225) assays and by Qubit Fluorometer (Invitrogen) according to the manufacturers’ recommendations.

### SDS-PAGE and Western Blot Analysis

Isolated uEVs were solubilized in sample buffer and subjected to SDS-PAGE and Western blotting. The primary antibodies anti-CD63, and -CD9 were from SBI System Biosciences, anti-TSG101 was from Abcam (Ab83, 4A10), anti-CD81 was from Santa Cruz (Sc-166026), secondary anti-mouse (NA931) and anti-rabbit (NA934) HRP conjugated ECL antibodies were from GE Healthcare Life Sciences.

### Nanoparticle tracking analysis (NTA)

uEVs were investigated on the NanoSight NS300 Instrument (405 nm laser diode) according to the manufacturer’s protocols (Malvern Instruments Inc.). The instrument was calibrated prior to each experimental run for nanoparticle size and quantity using standardized nanoparticle dilutions provided by the manufacturer. Suitable dilution of isolated uEVs was defined before each measurement. Briefly, uEVs preparations were homogenized by vortexing followed by serial dilution (1:1000 to 1:10,000) in sterile PBS and analysed by NanoSight NS300. Each experimental run was performed in quintuplicate (each capture 60 s) and PBS used to assess background. For each sample, nanoparticle size distribution curve, refractive index, the relative nanoparticle concentration of a particular size was recorded, with the cumulative percentage of nanoparticles. NTA 2.3 software was used to analyse the data (the script: SETTEMP 25; CAMERAON; CAMERAGAIN 13; CAMERALEVEL 12; REPEATSTART; SYRINGLOAD 100; DELAY 10; SYRINGSTOP; DELAY 15; CAPTURE 60; DELAY 1; REPEAT 4; SETTEMP OFF; PROCESSINGLESETTING; EXPORTRESULTS).

### Electron microscopy

The uEV samples were imaged by transmission electron microscopy (TEM) with negative staining. uEV suspension (4 μl) of was applied on Formvar-coated and carbon stabilized copper grids (Plano) and stained with Nano-W (Nanoprobes, #2018) or NanVan (Nanoprobes, #2011). Samples were examined with Tecnai Spirit electron microscope (FEI, The Netherlands, Olympus-SIS Veleta CCD Camera, Cathode Tungsten/LaB6, Illumination:120 kV operated at 80 kV, Objective BioTWIN lens) and images were recorded using TIA/SerialEM Software.

### miRNA isolation from total urine

The Norgen Biotek’s Urine Cell-Free Circulating RNA Purification Maxi Kit (Cat. 56600) was used to isolate miRNAs from 50 ml urine samples according to manufacturer’s instructions.

### miRNA isolation from exosomes

The mirVana miRNA Isolation Kit (Life technologies, AM1560) was used for miRNA isolation from the urinary exosomes. Exosomes were lysed in the equal volume of sample/lysis buffer and further processed according to manufacturer’s protocol.

### Assessing RNA quantity and quality

RNA concentration and quality were assessed using Nanodrop 1000 spectrophotometer (Thermo Scientific), and Agilent RNA 6000 Pico Kit, Agilent RNA 6000 Nano Kit, Agilent Small RNA kit on Agilent 2100 Bioanalyzer (Agilent Technologies). For samples used in NanoString assays, Qubit fluorometer (Life Technologies), Qubit DNA BR Assay kit and Qubit Protein Assay kits were employed.

### cDNA synthesis and qPCR

TaqMan Advanced miRNA cDNA Synthesis Kit (Applied Biosystems, A28007) was used according to manufacturer’s instructions. MicroRNA advanced TaqMan assays were used for qPCR and results analysed with web browser-based Applied Biosystems™ Real-Time PCR Analysis Modules.

### NanoString nCounter analysis

miRNA presence and content of the samples were analysed with the nCounter Human miRNA Expression Assay kit (NanoString, Seattle, WA) according to manufacturer’s instructions with some modifications. Briefly, 3 µl of each total RNA sample was used as input into the nCounter Human miRNA sample preparation. Hybridization was conducted for 12 h at 65 °C. Subsequently, the strip tubes were placed into the nCounter Prep Station for automated sample purification and subsequent reporter capture. Each sample was scanned for 600 FOV on the nCounter Digital Analyzer. Three R Packages were used for NanoString Data Analysis: “NanoStringNorm” and “NanoStringDiff” in (Available in CRAN) to do positive and negative control subtraction and normalization and build the dataset and then “EdgeR” for expression profiling. We also used inbuilt R commands for heatmap generation and “Venny” for venn diagrams.

### Statistical analysis

Hierarchical clustering and the associated heatmaps for miRNA profiling data was generated with the function heatmap2 in the R package gplots or GENE-E R package. We used pairwise correlation matrix between items based on Pearson correlation method. The correlation matrix was converted as a distance matrix. Finally, clustering was calculated on the resulting distance matrix. We used average linkage method to calculate the distance matrix and “Venny” to draw simple Venn diagrams without size adjustments. To choose reference (endogenous control) miRNA, expression stabilities of the different miRNAs was evaluated using NormFinder R package. Normalized values were centred and scaled. Then normalized Ct values were used to create hierarchical clustering and heatmap. A one-way analysis of variance (ANOVA) was used to determine statistically significant differences between the means of two or more independent group exists. Tukey’s multiple comparisons and Sidak’s multiple comparisons tests were used to correct P values. The P value < 0.05 was considered as statistically significant (GraphPad Prism (version 7.0a)). Corrplot package in R was used to find the correlation between the chemical composition of urine, NTA results, protein quantification and RNA analysis. We computed the p-value of Pearson correlations using a custom R function and only show p-values < 0.05. Volcano graph was made by plotting -log10 adjusted p value of miRNA content in urinary exosomes compared to total urine against log2 fold change of corresponding samples using a custom R function.

## Electronic supplementary material

Dataset 1

Dataset 2
