# Supplementary material for: Improved isolation strategies to increase the yield and purity of human urinary exosomes for biomarker discovery
Source: Sci Rep. 2018 Mar 2;8:3945. doi: 10.1038/s41598-018-22142-x (PMC5834546; doi:10.1038/s41598-018-22142-x)

**Supplementary Material**

**to**

**“Improved isolation strategies to increase the yield and purity of human urinary exosomes for biomarker discovery”**

**by**

Ali Hashemi Gheinani ^1^, Mike Vögeli ^1, 2^, Ulrich Baumgartner ^3^, Erik Vassella ^3^, Annette Draeger ^4^, Fiona C. Burkhard ^1,5^, and Katia Monastyrskaya ^1, 5 *^

^1^ Urology Research Laboratory, Department for BioMedical Research, University of Bern, Switzerland

^2^ medi Zentrum für medizinische Bildung, Biomedizinische Analytik HF

Max-Daetwyler-Platz 2, 3014 Bern, Switzerland

^3^ Institute of Pathology, University of Bern, Switzerland

^4^ Institute of Anatomy, University of Bern, Switzerland

^5^ Department of Urology, University Hospital, 3010 Bern, Switzerland

* Corresponding author:

K. Monastyrskaya - Urology Research Laboratory, Department for BioMedical Research, University of Bern, Switzerland Tel. +41 31 632 87 76; Fax: +41 31 632 05 51

E-mail address: [monastyk@dbmr.unibe.ch](mailto:monastyk@dbmr.unibe.ch)

**Supplementary Figure Legends:**

**Supplementary Figure S1. Different uEV isolation methods: protocol overview.**

250 ml of urine were pooled from 5 healthy males (20-55 years old, 50 ml each). Serial centrifugation was performed to remove cells, debris and protein aggregates. The pool was divided and uEVs isolated using five different methods simultaneously. The resulting uEVs were characterized by NTA, miRNA NanoString profiling, protein quantification and electron microscopy. All the urine samples were analysed for their chemical composition.

**Supplementary Figure S2. Size exclusion chromatography column set-up.** Chromatography column (0.7 cm diameter) was stacked with 2% cross-linked agarose gel filtration media (Sepharose CL-2B) to 13 cm height. UC pellet was resuspended in 500 μl PBS and placed on top of the Sepharose and a peristaltic pump was used to add the particle-free PBS via tubes on top of the SEC column. As the pump was pushing the buffer on the column, the droplets were collected in 50 x 0.5 ml fractions.

**Supplementary Figure** **S3**. **Comparison of protein measurement methods.**

Exosome-containing fractions of SEC were determined by NTA and subjected to different methods of protein quantification. Qubit method (green) reported higher amount of proteins compared to BCA (blue) and Bradford (red) in the same fractions.

**Supplementary Figure** **S4. Correlogram, all correlations**

The correlation between different parameters of urinalysis and uEVs. Positive correlations are displayed in blue and negative correlations in red colour. Colour intensity and the size of the circle are proportional to the correlation coefficients.

Supplementary Tables:

Supplementary Table ST1. Tukey's multiple comparisons test for 50-150 nm subpopulation of uEVs.

| **Tukey's multiple comparisons test** | **Corresponding mean of methods** | **Mean Diff.** | **Summary** | **Adjusted P value** |
| --- | --- | --- | --- | --- |
| **UC vs. UC-SEC** | 8.00E+08 vs. 6.29E+08 | 1.71E+08 | ns | 0.518 |
| **UC vs. C-SEC** | 8.00E+08 vs. 3.96E+08 | 4.04E+08 | * | 0.022 |
| **UC vs. PEG** | 8.00E+08 vs. 2.31E+07 | 7.77E+08 | *** | 0.000 |
| **UC vs. PEG-SEC** | 8.00E+08 vs. 7.79E+06 | 7.92E+08 | *** | 0.000 |
| **UC-SEC vs. C-SEC** | 6.29E+08 vs. 3.96E+08 | 2.33E+08 | ns | 0.250 |
| **UC-SEC vs. PEG** | 6.29E+08 vs. 2.31E+07 | 6.06E+08 | ** | 0.001 |
| **UC-SEC vs. PEG-SEC** | 6.29E+08 vs. 7.79E+06 | 6.22E+08 | ** | 0.001 |
| **C-SEC vs. PEG** | 3.96E+08 vs. 2.31E+07 | 3.73E+08 | * | 0.034 |
| **C-SEC vs. PEG-SEC** | 3.96E+08 vs. 7.79E+06 | 3.88E+08 | * | 0.027 |
| **PEG vs. PEG-SEC** | 2.31E+07 vs. 7.79E+06 | 1.53E+07 | ns | 1.000 |

| **Items** | **Sample#1** | **Sample#2** | **Sample#3** | **Sample#4** | **Sample#5** | **Sample#6** |
| --- | --- | --- | --- | --- | --- | --- |
| **Sodium (mmol/l)** | 87 | 106 | 116 | 116 | 137 | 133 |
| **Potassium (mmol/l)** | 31 | 27 | 38 | 46 | 51 | 39 |
| **Chloride (mmol/l)** | 65 | 55 | 76 | 79 | 129 | 119 |
| **Calcium (mmol/l)** | 3.3 | 3.4 | 3.46 | 4.27 | 4.71 | 3.48 |
| **pH** | 6.6 | 6.23 | 6.8 | 6.55 | 6.9 | 6.1 |
| **Zinc (mmol/l)** | 7.6 | 6.5 | 6.5 | 6.9 | 9.7 | 7.4 |
| **Pancreatic_amylase (U/l)** | 111 | 122 | 128 | 153 | 179 | 138 |
| **Inorganic._phosphate (U/l)** | 24 | 26 | 28.2 | 33.1 | 32.5 | 20.64 |
| **Glucose(mmol/l)** | 0.27 | 0.27 | 0.36 | 0.33 | 0.4 | 0.43 |
| **uric_acid** | 2222 | 2223 | 2518 | 3027 | 2456 | 2074 |
| **Protein (g/l)** | < 0.15 | < 0.15 | < 0.15 | < 0.15 | < 0.15 | < 0.15 |
| **Creatinine (umol/smp)** | 1.2.E+04 | 1.1.E+04 | 1.3.E+04 | 1.5.E+04 | 1.4.E+04 | 1.2.E+04 |
| **Albumin (mg/l)** | 0.25 | 0.25 | 0.3 | 0.39 | 0.25 | 0.25 |
| **osmolality** | 675 | 665 | 626 | 773 | 777 | 570 |
| **Urea (mmol/l)** | 333 | 322 | 280 | 388 | 342 | 198 |
| **magnesium(mmol/l)** | 5 | 5 | 6 | 6 | 4 | 3 |
| **Particle_count (particles)** | 4.4.E+11 | 5.5.E+12 | 8.9.E+12 | 5.9.E+12 | 5.8.E+12 | 5.3.E+12 |
| **Urine_RNA(Reads)** | 14'475 | 55'967 | 35'975 | 10'927 | 35'781 | 47'062 |
| **Exosome_RNA (Reads)** | 7'915 | 7'664 | 10'140 | 8'016 | 7'286 | 8'149 |
| **Protein_content (ug)** | 302 | 415 | 685 | 435 | 396 | 572 |

**Supplementary Table ST2.**  Urinanalysis of samples used for total RNA analysis and urinary exosome isolation

Figure S1


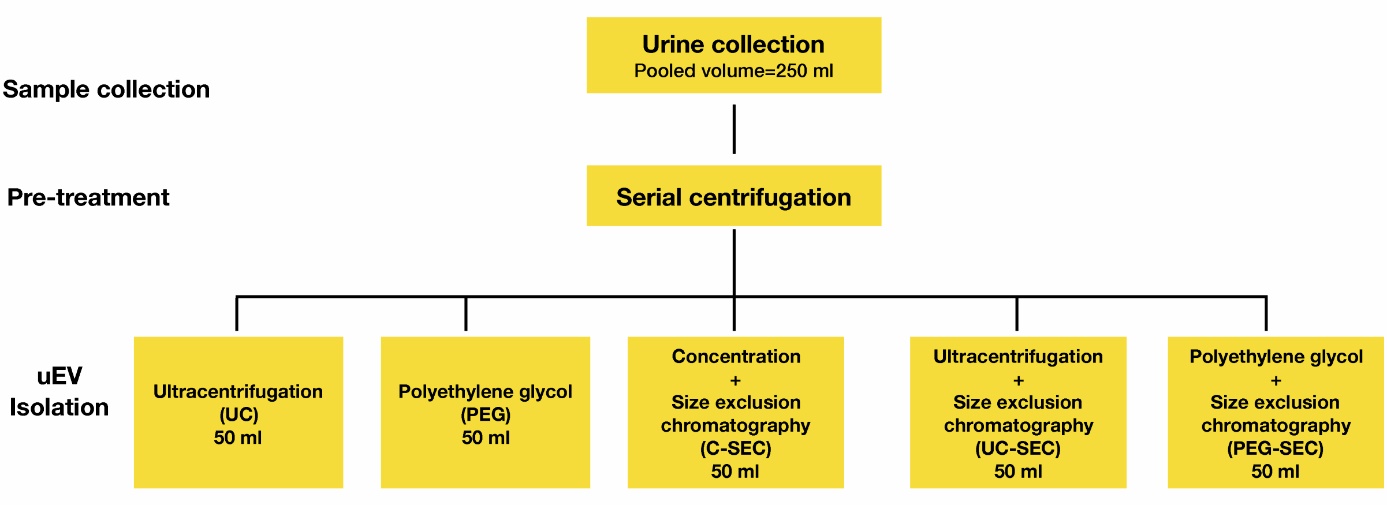


Figure S2


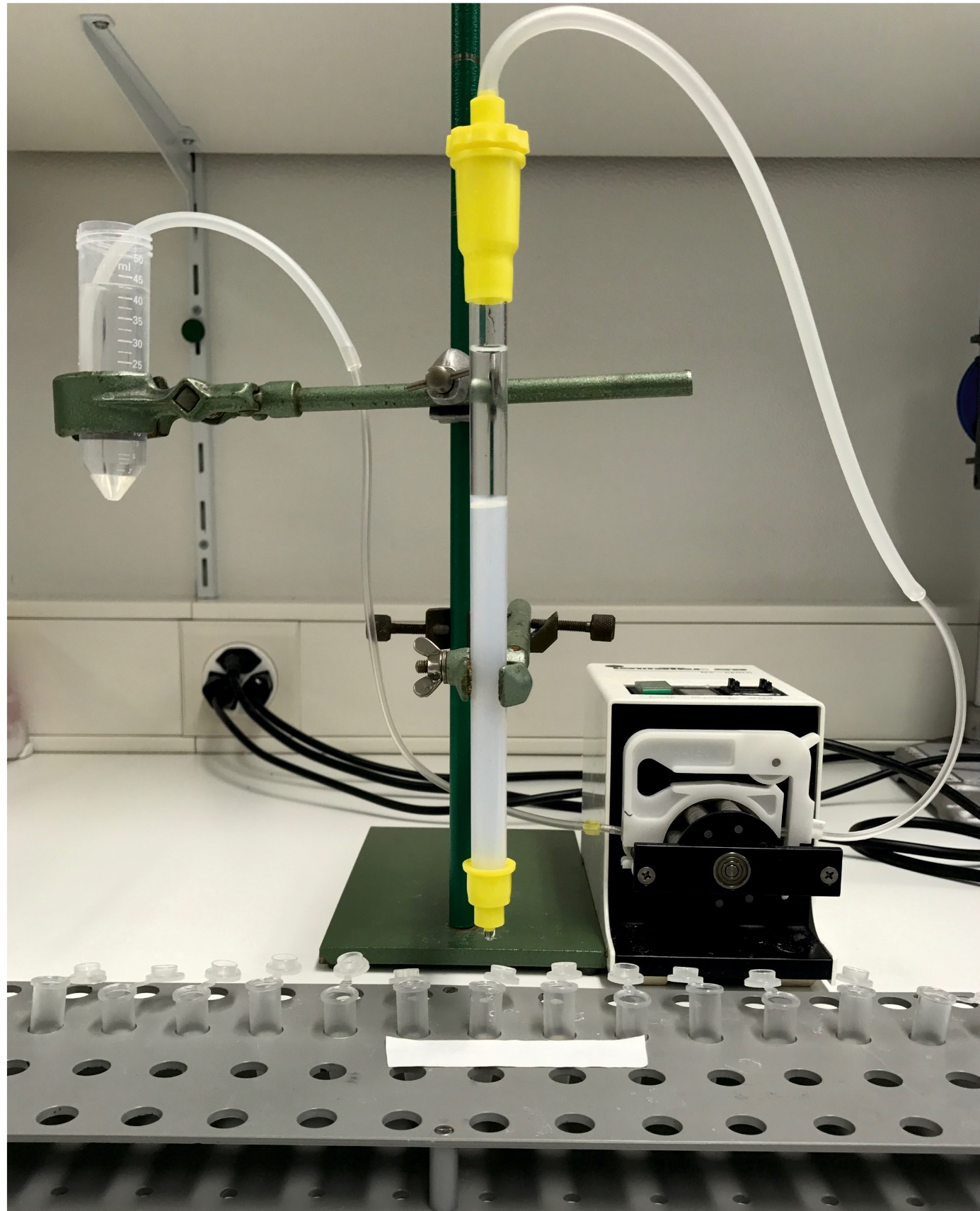


Figure S3


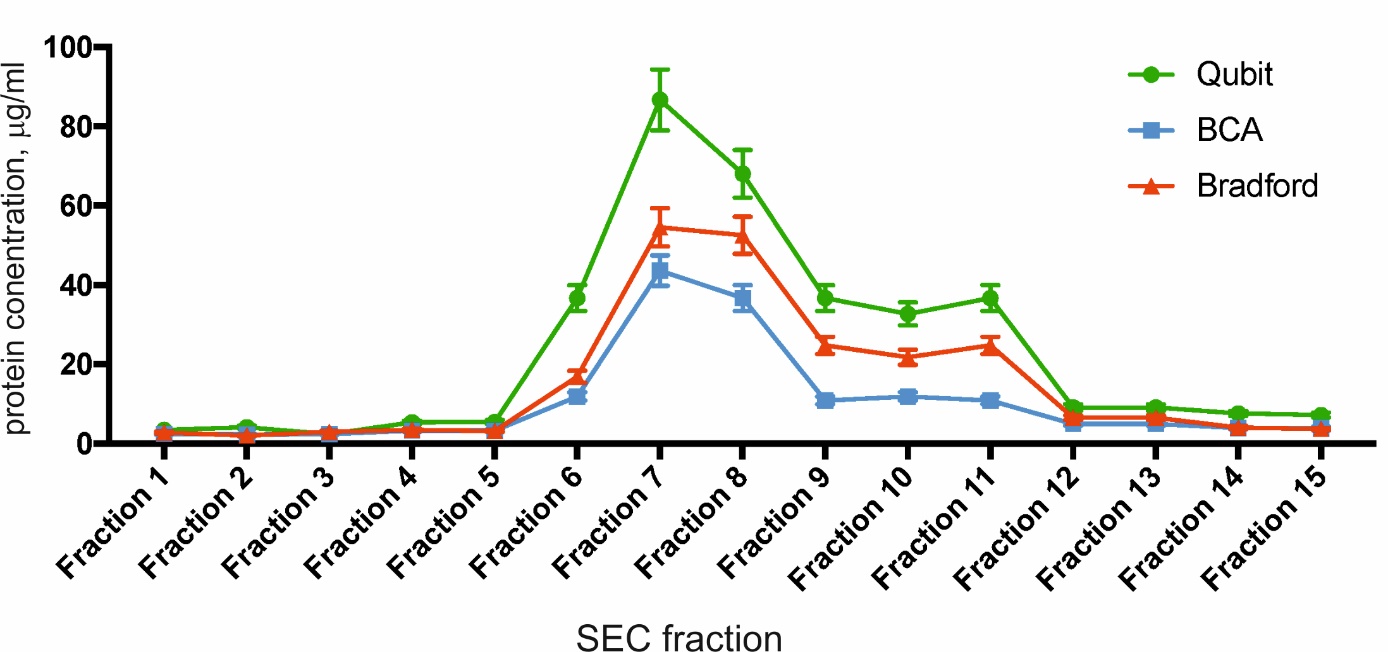


Figure S4


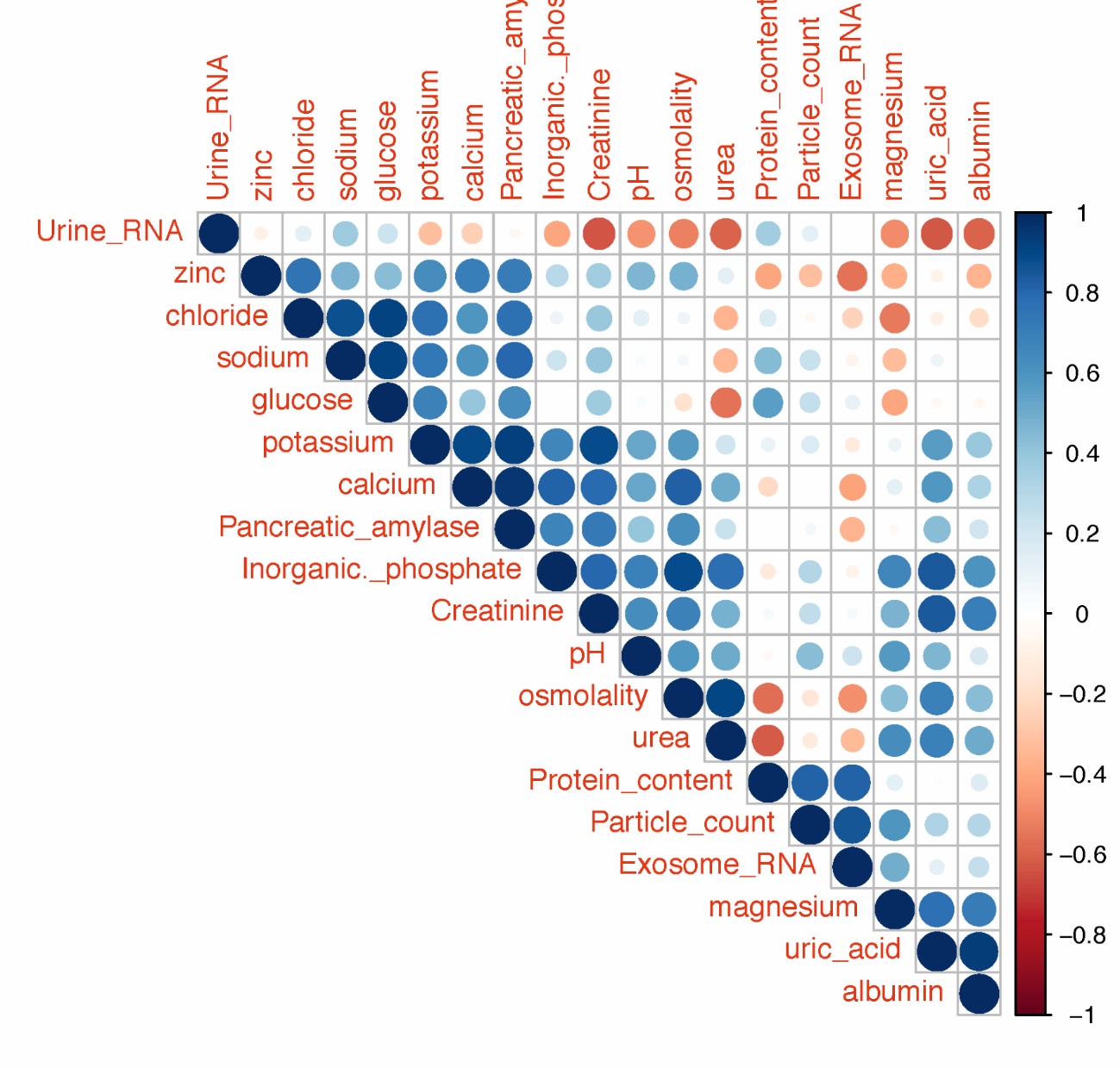

Supplement: Supplementary file 1 — Dataset 1 [file 41598_2018_22142_MOESM1_ESM.docx]
